# GaAs Nanowire Photodetectors Based on Au Nanoparticles Modification

**DOI:** 10.3390/ma16041735

**Published:** 2023-02-20

**Authors:** Fengyuan Lin, Jinzhi Cui, Zhihong Zhang, Zhipeng Wei, Xiaobing Hou, Bingheng Meng, Yanjun Liu, Jilong Tang, Kexue Li, Lei Liao, Qun Hao

**Affiliations:** 1 State Key Laboratory of High Power Semiconductor Lasers, Changchun University of Science and Technology, Changchun 130022, China; 2 Department of Electrical and Electronic Engineering, Southern University of Science and Technology, Shenzhen 518055, China; 3 State Key Laboratory for Chemo/Biosensing and Chemometrics, College of Semiconductors (College of Integrated Circuits), Hunan University, Changsha 410082, China; 4 School of Optoelectronics, Beijing Institute of Technology, Beijing 100081, China

**Keywords:** GaAs nanowires, photodetectors, Au nanoparticles, plasmons, Schottky barriers

## Abstract

A high-performance GaAs nanowire photodetector was fabricated based on the modification of Au nanoparticles (NPs). Au nanoparticles prepared by thermal evaporation were used to modify the defects on the surface of GaAs nanowires. Plasmons and Schottky barriers were also introduced on the surface of the GaAs nanowires, to enhance their light absorption and promote the separation of carriers inside the GaAs nanowires. The research results show that under the appropriate modification time, the dark current of GaAs nanowire photodetectors was reduced. In addition, photocurrent photodetectors increased from 2.39 × 10^−10^ A to 1.26 × 10^−9^ A. The responsivity of GaAs nanowire photodetectors correspondingly increased from 0.569 A∙W^−1^ to 3.047 A∙W^−1^. The reasons for the improvement of the photodetectors’ performance after modification were analyzed through the energy band theory model. This work proposes a new method to improve the performance of GaAs nanowire photodetectors.

## 1. Introduction

Photodetectors employing advanced material systems and device designs have attracted significant attention, from the original photovoltaic effect to current intelligent optoelectronic sensors [1,2,3,4,5,6,7]. Under the requirement for miniaturization of optoelectronic devices, GaAs nanowires stand out because of their higher carrier mobility and absorption coefficients compared with traditional low-dimensional materials [8,9]. In addition to these properties, they have a direct band gap (1.42 eV) and high optical sensitivity, which makes them a potential material for the preparation of room temperature visible-infrared photodetectors [10,11,12]. However, the high surface state density of GaAs nanowires usually leads to a large dark current in photodetectors, which seriously affects the device responsivity [13,14,15]. Recently, the relevant reports have proposed that the surface state in GaAs nanowires can be improved by sulfur passivation, thereby reducing the dark current of photodetectors [16,17]. However, the present researches remain major challenges specially on how to effectively enhance the photocurrent of GaAs nanowire photodetectors [18].

Generally, Au, Ag, Cu and other metal group nanostructures manifest significant light absorption and conversion properties owing to their intrinsic localized surface plasmon resonance (LSPR) [19,20,21,22,23,24]. Thus, the utilization of the LSPR effect of metal nanostructures is an effective strategy in improving the absorption of semiconductor materials at visible–near-infrared wavelengths [25,26,27]. Previous studies have shown that the nanowires can be modified by Au colloidal solution deposition and Au nanoparticle chemical generation [28,29]. However, both the preparation of Au colloidal solutions and the growth of Au nanoparticles on nanowires usually have high requirements on the ratio of reactants and reaction conditions. A simple and efficient design for modifying GaAs nanowires using Au nanoparticles is urgently needed to improve the performance of nanowire photodetectors [30].

Herein, we successfully fabricate a GaAs nanowire photodetector with Au nanoparticle modification, with only a brief regulation for the evaporation rate and time in the thermal evaporation system. The key in the modification process of GaAs nanowires is to properly adjust the diameter and number of Au nanoparticles by controlling the evaporation rate time, respectively, to effectively amplify the photocurrent. The reason for promoting the photoelectric detection performance is the coupling of the electron gas in the Au nanoparticles with the excitation light [31,32,33,34,35]. Furthermore, the metal particles on the surface of the GaAs nanowires will introduce a local Schottky barrier, which generates a space charge region which promotes the separation and collection of photogenerated carriers effectively [36,37,38,39]. This work opens up an avenue for constructing high-performance photodetectors, showing considerable potential in miniaturized optoelectronic devices.

## 2. Materials and Methods

### 2.1. Growth of GaAs Nanowires

GaAs nanowires were grown using the Ga-assisted self-catalyzed method. The MBE system model was DCA P600, and the substrate used was an n-type Si(111) substrate. First, the Si substrate was sonicated with acetone, absolute ethanol and deionized water for 5 min each to remove surface impurities. Subsequently, the substrate was transferred to the preparation chamber and degassed at 400 °C, then transferred to the growth chamber to deposit Ga droplets at 620 °C for 26 s. The formal growth of GaAs nanowires started after a growth pause of 80 s. During the growth process, the temperature was kept constant at 620 °C and the V/III beam current ratio was kept constant at 25.8 (the Ga beam was set to 6.2 × 10^−8^ Torr, the As beam current was set to 1.6 × 10^−6^ Torr and 1 Torr = 133.322 Pa). After 2 h of growth, the Ga source was first turned off and the As source was kept on until the substrate temperature was cooled to 300 °C. Finally, the growth of the GaAs nanowires was completed when the substrate temperature cooled to room temperature.

### 2.2. Fabricate Nanowire Photodetectors

To fabricate GaAs nanowire photodetectors, the grown GaAs nanowire arrays were transferred onto a P-type Si substrate containing 100 nm SiO_2_ by a mechanical transfer method. Cr/Au (10 nm/50 nm) electrodes were prepared by electron beam lithography (EBL), metal thermal evaporation and lift-off processes. With the aim of pursuing better metal-semiconductor contact, GaAs nanowires were immersed in 10% hydrochloric acid before the metal electrode was evaporated to the ends of the GaAs nanowires, which could remove the natural oxide layer on the surface of the GaAs nanowires [40,41]. The photoelectric properties of GaAs nanowire photodetectors were tested using the KEYSIGHT B1500 semiconductor analyzer under vacuum and at room temperature.

## 3. Results and Discussion

**Figure 1:**
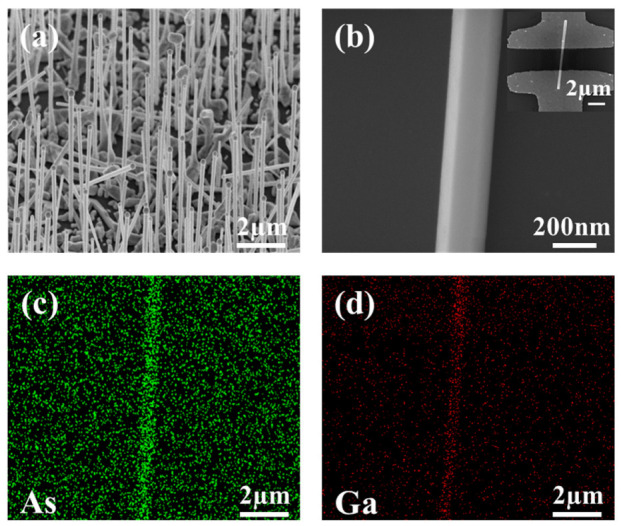
(**a**) SEM images of a GaAs nanowire array (**a**) and single GaAs nanowire (**b**). The illustration in the upper right corner is SEM diagram of GaAs nanowire photodetectors. (**c**,**d**) Energy dispersive X-ray spectroscopy (EDX) of the single GaAs nanowire.

SEM images of the MBE-grown GaAs nanowire array and a single nanowire are illustrated in Figure 1a,b. It is clearly shown that the average length of a GaAs nanowire is about 6 μm and the diameter is about 180 nm, respectively. Energy dispersive X-ray spectroscopy (EDX) analysis was employed to assess the chemical composition of the fabricated GaAs nanowire structure. As shown in Figure 1c,d, the EDX mappings reveal the homogeneous spatial distribution of elements Ga and As within the GaAs nanowires. The right-corner inset in Figure 1b presents the SEM image of the GaAs nanowire photodetector structure, with a channel length of 2 μm.

**Figure 2:**
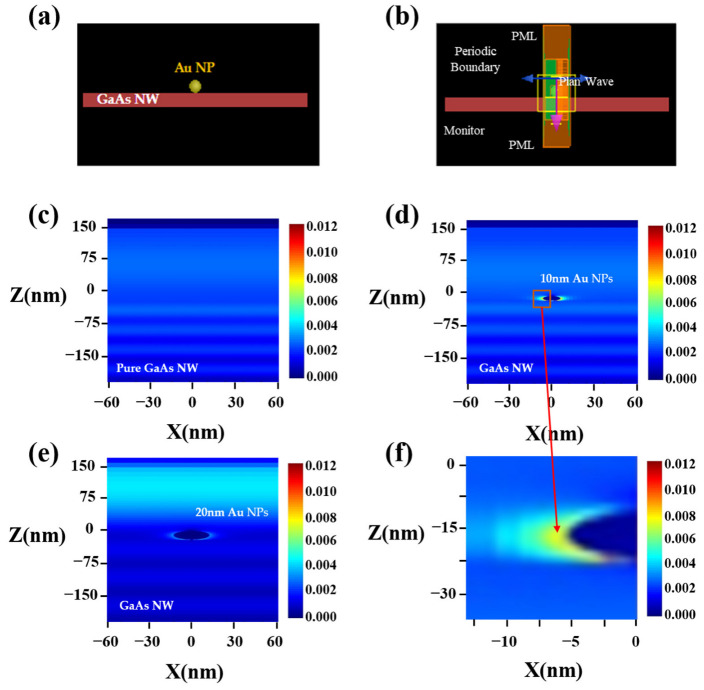
Simulation results by FDTD. (**a**,**b**) The structure of GaAs nanowire photodetectors decorated with Au nanoparticles. (**c**) Absorbed optical field distribution of pristine GaAs nanowires under the excitation light of 532 nm. (**d**,**e**) Distribution of absorption light field after modified GaAs nanowires by Au nanoparticles with the diameter of 10 nm, 20 nm. (**f**) Absorbed optical field intensity distribution at 1.77 nm to the left of Au nanoparticle.

To verify the enhancement of Au nanoparticles for the light absorption of GaAs nanowires, the finite difference time domain (FDTD) on the absorb optical field intensity distribution of different models are simulated in Figure 2. Considering the random distribution of Au nanoparticles attached to the nanowires, we only studied the light-field effect of a single particle, and simulated the random effect of Au particles on the entire nanowire under the light field by the periodic boundary conditions. The diameter of nanowires was 200 nm, and the size of nanoparticles were 10 nm and 20 nm. A plane wave entered the nanowires vertically as the incident light source and monitors were added at different positions to analyze the field changes in Figure 2a,b. The excitation light source was set as 532 nm to illuminate these different models. Figure 2c shows the absorbed optical field intensity distribution of a single GaAs nanowire. After modifying Au nanoparticles with a diameter of 10 nm on the surface of GaAs nanowires, it can be clearly observed that absorbed optical field intensity increased at the junction of the Au nanoparticles and GaAs nanowires in Figure 2d. In addition, we also used different diameter Au nanoparticles to modify the GaAs nanowire surface in Figure 2e. When the diameter of the Au nanoparticles was regulated to 20 nm, the absorption light field intensity at this time was reduced compared to the counterparts with a diameter of 10 nm. These results are consistent with previous findings by other researchers [42]. The greater the difference between the wavelength of the excitation light and the diameter of the metal particle, the greater the coupling range between the electron gas in the metal particle and the excitation light. In addition, we wanted to further understand the specific multiples of the intensity of the absorbed optical field at the junction before and after modification. Figure 2f shows the absorbed optical field intensity distribution at 1.77 nm to the left of Au nanoparticles. The strength of the optical field here is 4 times that of the same position on the original GaAs nanowires. In summary, the simulation results can clearly demonstrate the influence of Au nanoparticles on the performance of GaAs nanowires, which will lay a good foundation for our subsequent experiments.

**Figure 3:**
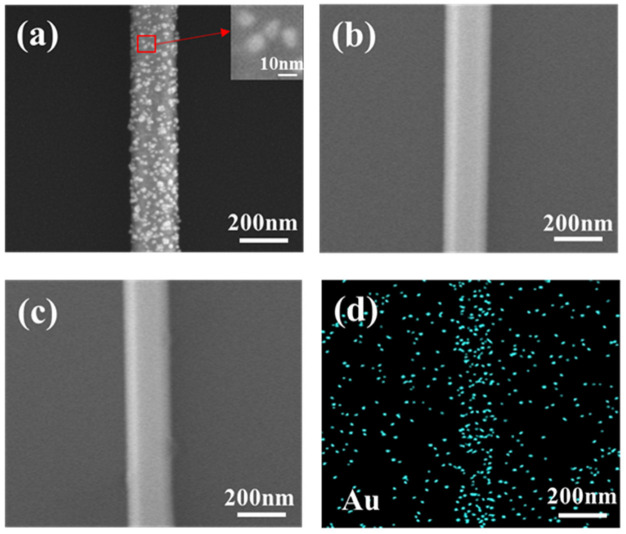
(**a**–**c**) SEM images of GaAs nanowires under modification time of 10 s, 30 s, 50 s, respectively. (**d**) Energy dispersive X-ray spectroscopy (EDX) of Au nanoparticles under modification time of 10 s.

GaAs nanowire photodetectors were modified using Au nanoparticles. The preparation principle was that solid gold will melt under high temperature and pressure, Au nanoparticles will evaporate to GaAs nanowire, and the surface of GaAs nanowires in GaAs nanowire photodetectors channel will be attached by the Au nanoparticles. The size and quantity of the Au nanoparticles were adjusted by regulating the evaporation rate and time. As mentioned above, in order to improve the performance of GaAs nanowire photodetectors, we were committed to preparing Au nanoparticles with a diameter of 10 nm to modify the GaAs nanowires. We set the evaporation rate of the thermal evaporation system to a minimum rate of 0.1 Å/s, which ensures the smallest diameter for Au nanoparticles in the preparation process. In order to find the appropriate time to modify the GaAs nanowires in the channel of photodetectors, we prepared three GaAs nanowire photodetectors and modified the GaAs nanowires in the channel, with modification times of 10 s, 30 s and 50 s, respectively. Figure 3a–c shows SEM images of modified GaAs nanowires at modification times of 10 s, 30 s and 50 s, respectively. An SEM image of the Au nanoparticle structure is shown in the upper right corner of Figure 3a. When the modification time was 10 s, Au nanoparticles were randomly distributed on the surface of GaAs nanowires. Particularly, the average diameter of Au nanoparticles was about 10 nm, which perfectly meets our previous expectations. When the modification time was increased to 30 s and 50 s, Au nanoparticles evenly covered the surface of GaAs nanowires. This caused us to not be able to distinguish the individual Au nanoparticles. To further observe the composition of Au nanoparticles on the surface of GaAs nanowires, energy dispersive X-ray spectroscopy (EDX) of Au nanoparticles of the samples was measured under a modification time of 10 s, as shown in Figure 3d.

**Figure 4:**
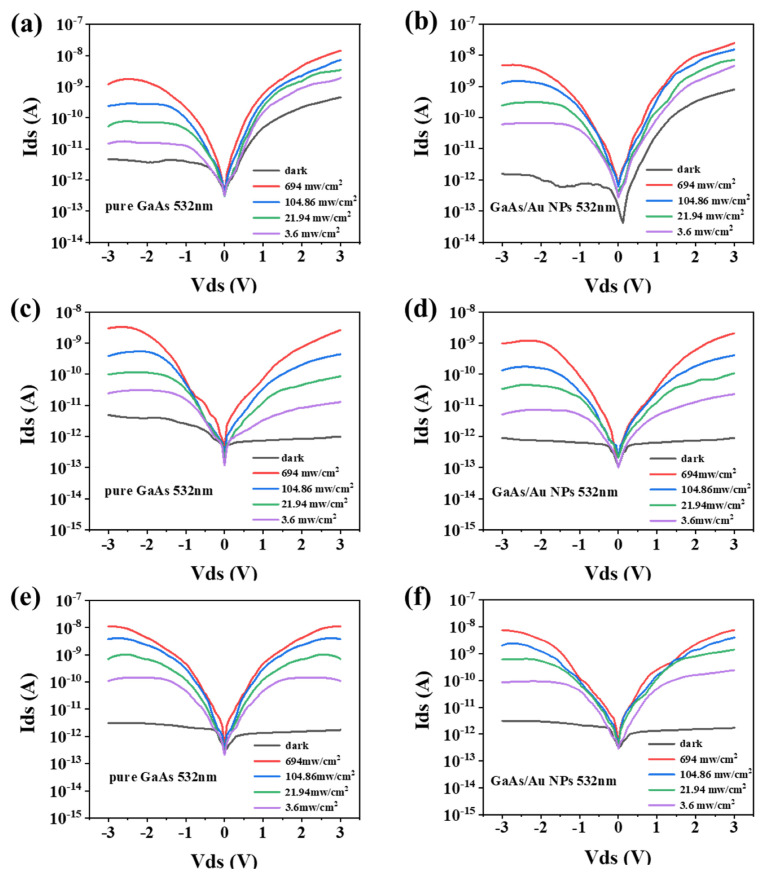
I-V characteristic curves of the GaAs nanowire photodetectors before and after modification. (**a**,**c**,**e**) I-V characteristic curve of the original photodetectors. (**b**,**d**,**f**) I-V characteristic curve of the photodetectors after the modification time is 10 s, 30 s and 50 s, respectively.

The I-V characteristic curves of the GaAs nanowire photodetectors with different modification times and their corresponding unmodified devices under a 532 nm excitation light are shown in Figure 4. By comparing Figure 4a,b, when the modification time was 10 s, the dark current of GaAs nanowire photodetectors decreased from 4.60 × 10^−12^ A to 1.56 × 10^−12^ A under the optical power density of 104.86 mw∙cm^−2^. The reduction of dark current was attributed to the improved surface state of GaAs nanowires. Meanwhile, the photocurrent of GaAs nanowire photodetectors increased from 2.39 × 10^−10^ A to 1.26 × 10^−9^ A. The improvement of the photocurrent can be attributed to the introduction of localized plasmons and surface Schottky barriers, which further enhance the light absorption of GaAs nanowires and promote the separation of photogenerated carriers inside GaAs nanowires. Figure 4c,d displays the changes in the performance of the photodetectors before and after 30 s modification time. It can be intuitively found that the modification time of 30 s not only reduces the dark current, but also decreases the photocurrent of GaAs nanowire photodetectors. The reason for the decrease in the photocurrent of the GaAs nanowire photodetectors is that too many Au nanoparticles are evenly covered on the surface of the GaAs nanowires, which will partially reflect the excitation light, resulting in the decrease in the light absorption rate. Similarly, when the modification time was 50 s, the dark current and photocurrent of the modified photodetectors were both reduced, as shown in Figure 4e,f. Based on these results, we believe that modification time is critical in the performance of GaAs nanowire photodetectors. Additionally, a suitable modification time (10 s) is acquired to increase the photocurrent of GaAs nanowire photodetectors.

**Figure 5:**
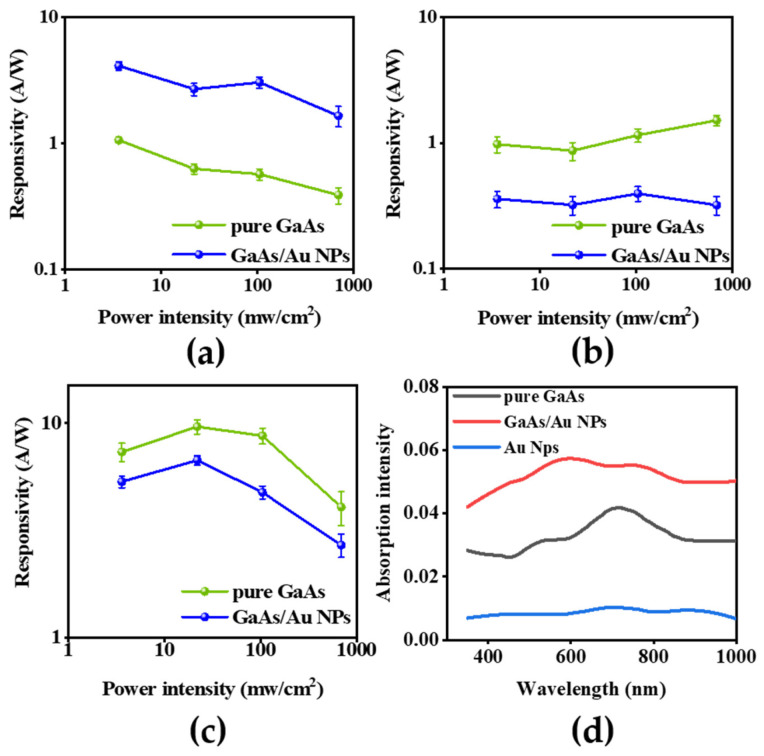
Responsivity comparison of photodetectors before and after Au nanoparticles modification, respectively correspond to modification time of 10 s (**a**), 30 s (**b**) and 50 s (**c**). (**d**) Comparison of absorption spectra before and after modification, the modification time is 10 s.

The responsivity is also an important physical parameter describing the photoelectric conversion of GaAs nanowire photodetectors [37], which can be expressed as

(1) $$R=\frac{I_{light}-I_{dark}}{A*P}$$

where *A* represents the photosensitive area and *P* is the optical power density (mw∙cm^−2^) [43,44,45,46]. When the modification time was 10 s, the responsivity of GaAs nanowire photodetectors improved under different optical power densities, as shown in Figure 5a. Obviously, under the optical power density of 104.86 mW∙cm^−2^, the responsivity of GaAs nanowire photodetectors increased from 0.569 A∙W^−1^ to 3.047 A∙W^−1^. Figure 5b,c show that the responsivity of GaAs nanowire photodetectors decreases under different optical power densities. To further learn the intrinsic mechanism of the performance improvement of GaAs nanowire photodetectors under the modification time of 10 s, we compared the absorption spectral intensity of GaAs nanowires, Au nanoparticles and GaAs nanowire/Au nanoparticles. Figure 5d shows the UV-IR absorption spectra of GaAs nanowire photodetectors before and after modification. It can be observed that the absorption of the modified photodetectors under the excitation light in the 350–1000 nm band is stronger than that of the original photodetectors. This is due to the mechanical oscillation of the electron gas in the Au nanoparticles under the action of the excitation light. At that moment, the electron gas in the Au nanoparticles is coupled with the excitation light to enhance the light absorption of GaAs nanowires. This is also a good explanation for the FDTD simulation results presented earlier. As mentioned above, we determined that Au nanoparticles could enhance the light absorption rate of GaAs nanowires from two aspects (simulation results and specific experimental results), which can improve the photocurrent of GaAs nanowire photodetectors.

We also compared the performances of various GaAs-based photodetectors in previous reports (Table 1). Notably, our GaAs NW/Au NPs photodetector not only realized the doubling of the dark current but also increases the photocurrent synchronization by one order of magnitude, which endowed the photodetector with ultra-high responsiveness. Additionally, its responsiveness was almost higher than that of other reported GaAs-based nanowire photodetectors.

**Figure 6:**
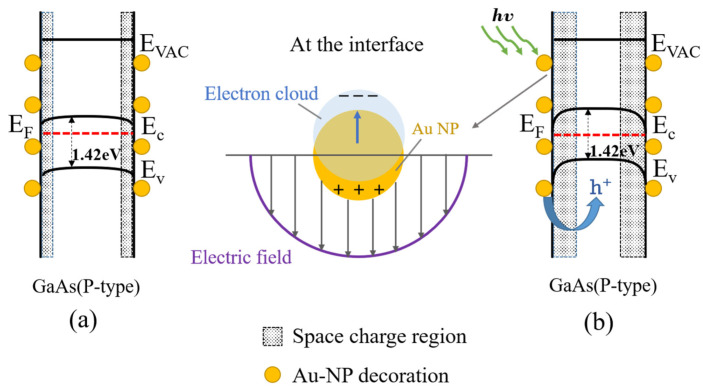
(**a**) Band structure diagram of Au nanoparticle-modified GaAs nanowires. (**b**) Excitation light excites the electron gas inside Au nanoparticles to oscillate upward to enhance the space charge region.

The enhancement of the photocurrent can be attributed to the formation of the surface Schottky barrier displayed in Figure 6a,b. Since the work function of Au (φ_Au_ = 5.1 eV) is larger than that of GaAs nanowires (φ_GaAs_ = 4.78 eV) [49,50,51], the electrons at the interface between the GaAs nanowires and Au nanoparticles are more likely to flow to the inside of the Au nanoparticles. The outer negative and inner positive space charge regions are formed at the surface of GaAs nanowires. When the excitation light source irradiates Au nanoparticles, Au nanoparticles absorb light energy and produce hot carriers. The hot holes flow to GaAs nanowires, which improves the photocurrent of the GaAs nanowire photodetectors and enhances the photoresponse under the applied bias [47,48].

## 4. Conclusions

A high-performance GaAs nanowire photodetector based on Au nanoparticle modification was prepared in this paper. The main advantage of this paper was that Au nanoparticles were attached to nanowires by thermal evaporation to improve the performance of the photodetectors. The FDTD simulation results were consistent with the proposed results, that the introduction of Au nanoparticles would increase the electromagnetic field amplitude in the semiconductor. Additionally, it was found that the optimal time for Au nanoparticles to modify photodetectors was 10 s. Under this condition, the photocurrent of GaAs nanowire photodetectors increased from 2.39 × 10^−10^ A to 1.26 × 10^−9^ A under the 532 nm excitation light. Moreover, under the optical power density of 104.86 mW∙cm^−2^, the responsivity of GaAs nanowire photodetectors increased from 0.569 A∙W^−1^ to 3.047 A∙W^−1^. This is because of the fact that the electron gas in the Au nanoparticles is excited to generate oscillations. The coupling of a part of the electron gas and the external electromagnetic wave enhances the light absorption efficiency of GaAs nanowire photodetectors. The hot holes flow to GaAs nanowires, which improves the photocurrent of GaAs nanowire photodetectors and enhances the photoresponse under the applied bias. This study proposes a simple and efficient method for improving the performance of photodetectors.

## Figures and Tables

**Table 1:** Performance comparison of GaAs-based NW photodetectors.

Material	*I_dark_* (A)	*I_light_* (A)	*R* (A∙W^−1^)	Ref
Commercial GaAs	/	/	0.45	[16]
GaAsSb	3 × 10^−7^	5 × 10^−7^	2.37	[47]
GaAs/GaAsAl quantum well NW	1.2 × 10^−12^	1.35 × 10^−11^	0.199	[48]
GaAs NW, surface passivation	7.18 × 10^−14^	1.8 × 10^−10^	18.2 to 25	[16]
GaAs NW-Au NPs	6.9 × 10^−13^	2.92 × 10^−12^	6.56	[18]
GaAs NW	4.84 × 10^−12^	2.39 × 10^−10^	0.569	This work
GaAs NW-Au NPs	1.95 × 10^−12^	1.28 × 10^−9^	3.047	This work

## Data Availability

Not applicable.
